# Exercise training with dietary counselling increases mitochondrial chaperone expression in middle-aged subjects with impaired glucose tolerance

**DOI:** 10.1186/1472-6823-8-3

**Published:** 2008-03-27

**Authors:** Mika Venojärvi, Sirkka Aunola, Raivo Puhke, Jukka Marniemi, Helena Hämäläinen, Jukka-Pekka Halonen, Jaana Lindström, Merja Rastas, Kirsti Hällsten, Pirjo Nuutila, Osmo Hänninen, Mustafa Atalay

**Affiliations:** 1 Institute of Biomedicine, Physiology, University of Kuopio, POB 1627, FI-70211, Kuopio, Finland; 2 Medical Laboratory Technology, Turku University of Applied Sciences, Ruiskatu 8, FI-20721, Turku, Finland; 3 Department of Health and Functional Capacity, Laboratory for Population Research, National Public Health Institute, Turku, Finland; 4 Institute of Exercise Biology and Physiotherapy, University of Tartu, Tartu, Estonia; 5 Research Department, Social Insurance Institution, Turku, Finland; 6 Department of Health Promotion and Chronic Disease Prevention, Diabetes Unit, National Public Health Institute, Helsinki, Finland; 7 Department of Health Promotion and Chronic Disease Prevention, Nutrition Unit, National Public Health Institute, Helsinki, Finland; 8 Turku PET Centre, University of Turku, Turku, Finland

## Abstract

**Background:**

Insulin resistance and diabetes are associated with increased oxidative stress and impairment of cellular defence systems. Our purpose was to investigate the interaction between glucose metabolism, antioxidative capacity and heat shock protein (HSP) defence in different skeletal muscle phenotypes among middle-aged obese subjects during a long-term exercise and dietary intervention. As a sub-study of the Finnish Diabetes Prevention Study (DPS), 22 persons with impaired glucose tolerance (IGT) taking part in the intervention volunteered to give samples from the *vastus lateralis *muscle. Subjects were divided into two sub-groups (IGTslow and IGTfast) on the basis of their baseline myosin heavy chain profile. Glucose metabolism, oxidative stress and HSP expressions were measured before and after the 2-year intervention.

**Results:**

Exercise training, combined with dietary counselling, increased the expression of mitochondrial chaperones HSP60 and glucose-regulated protein 75 (GRP75) in the *vastus lateralis *muscle in the IGTslow group and that of HSP60 in the IGTfast group. In cytoplasmic chaperones HSP72 or HSP90 no changes took place. In the IGTslow group, a significant positive correlation between the increased muscle content of HSP60 and the oxygen radical absorbing capacity values and, in the IGTfast group, between the improved VO_2max _value and the increased protein expression of GRP75 were found. Serum uric acid concentrations decreased in both sub-groups and serum protein carbonyl concentrations decreased in the IGTfast group.

**Conclusion:**

The 2-year intervention up-regulated mitochondrial HSP expressions in middle-aged subjects with impaired glucose tolerance. These improvements, however, were not correlated directly with enhanced glucose tolerance.

## Background

Diabetes and its complications are increasing as major causes of mortality and morbidity in the developed countries [1]. Insulin resistance and diabetes are associated with increased oxidative stresmpaired cellular defence systems [2-4]. We have recently shown in rats that streptozotocin-induced diabetes (SID) increase oxidative stress and resulted in impaired heat shock protein (HSP) responses in liver and skeletal muscle tissue [2]. HSPs are a family of proteins that promote cell survival after a wide variety of environmental stresses. The most widely studied HSP family is the 70-kDA family, which contains the constitutive HSP73 and inducible HSP72 forms. HSP72 plays a central role in protein synthesis, translocation, folding and assembly/disassembly of multimetric protein complexes as molecular chaperones [5]. In type 2 diabetic subjects, insulin resistance correlates with decreased expression of HSP72 in skeletal muscle [6]. HSP60 and glucose-regulated protein 75 (GRP75) are located in the mitochondria, where they are involved in the trafficking and processing of nuclear encoded peptides [7,8]. HSP90 is expressed in the cytosol, nucleus and endoplasmic reticulum [9] and has several physiological roles, including mediating tyrosine kinase receptor maturation and protein kinase B (PKB/AKT) stability, an important activator of glucose transports systems [9,10]. A number of studies have shown the expression of HSPs to vary depending on the muscle fibre type [11-13].

Oxidative stress, in which the increased production of reactive oxygen species (ROS) overwhelms endogenous antioxidant protection, may result in biomolecular damage. However, at lower concentrations, ROS also serve as secondary messengers, regulating cellular functions and adaptations. ROS have important role in signal transduction pathways involved in cell growt h, proliferation and differentiation, as the mitogen-activated protein kinase (MAPK) pathways [14] Oxidative stress may have an important role in the pathophysiology of insulin resistance and diabetes and its complications through increased oxidative damage, inflammation and apoptosis [15-17]. Recent *in vitro *and *in vivo *studies have also shown that the antioxidant supplementation suppresses ROS production and improves glucose tolerance and insulin sensitivity [18].

Strategies to decrease oxidative stress and to modulate HSP expression may have important implications for reducing insulin resistance and increasing the protection against diabetes and its complications. Very little is known about the effects of exercise and dietary interventions on the antioxidant defence and protection of HSPs in humans with impaired glucose metabolism [6,19], although information is available in different animal models [2]. Our purpose was to study whether a 2-year exercise and dietary intervention improves the antioxidative capacity and HSP defence of different skeletal muscle fibre phenotypes in middle-aged obese subjects with impaired glucose tolerance. Furthermore, we aimed to investigate the association of tissue defences with improved glucose metabolism.

## Methods

This study is a sub-study of the Finnish Diabetes Prevention Study (DPS), which has been described in detail elsewhere [20,21]. This sub-study was carried out in Turku with 22 subjects who volunteered to give muscle samples. The intervention started with intensive dietary counselling [20,22]. After 6 months, a supervised, progressive and individually tailored circuit-type resistance training program started. Blood samples for metabolic indices were taken at baseline and after 2 years and maximal exercise test and muscle sampling (with no heavy exercising during the preceding 2 days) were performed at 6 months and after 2 years. In skeletal muscle glucose metabolism, the greatest changes due to the dietary counselling were assumed to have occurred by 6 months. While this gives a good view of the changes in the regulation of the skeletal muscle metabolism induced by exercise, it does not allow us to distinguish the effects of either exercise or dietary counselling separately.

### Subjects

A total of 110 obese subjects with impaired glucose tolerance (IGT), were randomized to an intervention or a control group, based on two oral glucose tolerance tests. IGT was defined as 2-hour plasma glucose concentration from 7.8 to 11.0 mmol/l in oral glucose tolerance test (OGTT) with an intake of 75 g glucose in subjects whose fasting plasma glucose concentration was less than 7.8 mmol/l [23]. The test was repeated for subjects showing abnormal values, and the mean of the two 2-hour plasma glucose values was used as the inclusion criterion [20,22]. Twenty-two persons from the intervention group volunteered to give samples from their *vastus lateralis *muscle (n = 6 for females, n = 16 for males). Depending on the proportion of muscle myosin heavy chain II (MHC II) fibres, the subjects were divided into IGTslow group (less than 55% of MHC II fibres) or the IGTfast group [24]. The baseline characteristics of the subjects are shown in Table 1. The Ethical Committee of the Hospital District of South-West Finland, Turku, Finland and the Ethical Committee of the Rehabilitation Research Centre of the Social Insurance Institution of Finland approved the protocol of this sub-study. All subjects gave their written informed consent.

**Table 1:** Baseline characteristics of the subjects with impaired glucose tolerance (IGT) divided into the slow and fast fibre type sub-groups.

Characteristics	IGTslow	IGTfast
*n *= (Female/Male)	10 (4/6)	12 (2/10)
		
Age, yr	58.5 ± 2.0	54.1 ± 2.0
BMI (kg/m^2^)	29.9 ± 0.7	29.9 ± 0.7
Weight, kg	86.9 ± 2.8	92.0 ± 2.2
		
*Myosin heavy chain profile*		
MHC I, %	46.4 ± 2.9	30.6 ± 1.0***
MHC IIa, %	36.9 ± 3.5	47.2 ± 2.0**
MHC IIx, %	16.7 ± 3.3	22.2 ± 2.1
*Blood chemistry*		
Fp-glucose, mmol/l	6.0 ± 0.2	6.0 ± 0.1
2h-glucose, mmol/l	7.8 ± 0.3	7.5 ± 0.5
Fs-insulin, μU/ml	12.9 ± 1.0^a^	19.5 ± 2.7* ^b^
2 h-insulin, μU/ml	72 ± 13^a^	117 ± 35^b^
HbA_1c_, %	5.7 ± 0.1	5.7 ± 0.1
HOMA-IR	3.5 ± 0.3^a^	5.2 ± 0.7^b^

Data are given as means ± SE; * *P *< 0.05; ** P < 0.01, *** P < 0.001 ^a ^*n *= 9, ^b ^*n *= 11, Kruskall-Wallis test for difference between the groups.

### Intervention

The main goals of the intervention were as follows: 1) weight reduction of 5% or more, 2) less than 30% of the daily energy intake from fat, 3) less than 10% of the daily energy intake from saturated fat, 4) fiber intake 15 grams per 1000 kcal or more, and 5) moderately intense physical activity for 30 minutes per day or more. The implementation of the intervention program has been reported previously [20-22]. Briefly, the participants in the intervention group were given detailed and individualized counselling to achieve the set lifestyle goals. They had seven individual counselling sessions with the nutritionist during the first year and every three months thereafter. During the first 6 months, the intervention focused on dietary counselling, in order to allow the subjects to concentrate on changing their eating habits. The subjects were also individually encouraged by the nutritionist to increase their physical activity, and later on, to participate both in regular resistance training at gym and in aerobic exercise.

After 6 months, supervised exercise training twice a week was added to the intervention program [24]. The supervised training was progressive and individually designed, consisting mostly of strength and power training, interrupted by spinning exercises and aerobic gymnastic exercises. Power-type strength training at a fitness centre was performed as circuit training. Any physical activity lasting 30 min or longer was recorded by the participants. The subjects were advised to exercise with moderate-to-intensive effort for at least 30 min per session and three to four times a week. Eighteen of 22 participants attended to supervised training sessions at least once a week, but most of them twice or three times a week (range: 77–249 sessions) at a fitness centre. In addition, they trained by walking or other type of endurance exercises (range: 51–413 sessions). Only four participants did not attend regularly to the supervised training sessions, but they participated regularly in endurance type of sports or sports promoting muscular fitness or walking sessions (range: 105–554 sessions). Total amount of training varied from 105 to 584 sessions during the 2-year intervention period.

### Muscle biopsy

A skeletal muscle biopsy was taken under resting condition (no heavy exercising during the preceding 2 days) from the *vastus lateralis *muscle under local anaesthesia (lidocaine 10 mg/ml), using the 'semi-open' conchotomy technique [25]. Muscle samples were divided two separate pieces (approximately 40–60 mg) and immediately frozen in liquid nitrogen and stored at -70°C until analyzed. Samples for biochemical analyses were melted in an ice bath, weighed and homogenized in 1:50 (w/v) of 1 M Tris buffer pH adjusted to 7.5 in a manually operated all-glass homogenizer [26]. The enzyme activities[24] was measured immediately from the fresh homogenate and the rest of the homogenate were aliquots to five to six separate ependorf tubes and used for Western blot and ELISA analyses. The other piece of the muscle samples were used for the determination of myosin heavy chain profile.

### Exercise test and maximal oxygen uptake (VO_2max_)

A 2-min incremental cycle ergometer test until volitional exhaustion or fatigue of the lower limbs was employed to measure the maximal oxygen uptake (VO_2max_). The warm-up loading was 30–40 W in women and 40–60 W in men, depending on the age, size and physical fitness of the subjects. Thereafter, work rate was increased every 2nd min with equal increments (10–25 W) throughout the test. The increments were individually determined on the basis of the subject's physical fitness so that the maximum work rate would be reached in about 12–15 min [27]. Respiratory gas exchange was measured continuously by using a breath-by-breath method. VO_2max _was recorded as the highest averaged value over 30 s at the work rate maximum [27].

### Assessment of dietary intake

Nutrient intakes were assessed using a dietary analysis program developed by the National Public Health Institute [28]. At baseline and before the 24-month visit, the subjects were asked to complete a three-day food record [29,30].

### Determination of myosin heavy chain profile

The MHC isoform composition (MHC I, MHC IIa, MHC IIx) in muscle homogenate was determined by SDS-PAGE gel electrophoresis [31] by using a Bio-Rad Protean II Xi vertical slab gel system. The acrylamide and bis concentration were 4% in the stacking gel and 7.2% (w/vol) in the separating gel, and the gel matrix included 30% glycerol. Electrophoresis lasted 24 h at 120 V and +10°C. The gels were silver stained (Bio-Rad Silver stain Plus Kit, Hercules, CA, USA) and analyzed using computer-based image analysis system and software (Image Master 1D Elite, Amersham Pharmacia Biotech, Uppsala, Sweden).

### Western blot assays

Western blot assays were performed as described previously [2,32]. One-dimensional sodium dodecyl sulfate polyacrylamide (10%) gel electrophoresis was done to separate proteins according to their molecular weight. The blots were incubated overnight at +4°C with the following antibodies: anti-HSP60 (SPA-806), anti-HSP72 (SPA-810), anti-GRP75 (SPA-825), anti-HSP-90 (SPA-835) (Stressgen, Victoria, BC, Canada) and anti-4-HNE (210-767-R100) (Alexis Biochemical, San Diego, CA, USA). Horseradish peroxidase-conjugated immunoglobulins were used as secondary antibodies. Antibody binding was viewed by using an enhanced chemiluminescence method (NEN Life Science Products, Boston, MA, USA) and quantified by using the image analysis software (NIH-Image, MD, USA). The results were normalized according to beta-actin (LabVision/NeoMarkers, Fremont, CA, USA) values. The protein concentration of homogenates was measured by using BCA method (Pierce, Rockford, IL, USA).

### Elisa assay for protein carbonyls of skeletal muscle and serum samples

Protein carbonyls were measured with a slightly modified method according to Buss et al. [33] and Oksala et al. [34]. Briefly, protein derivation was carried out in 1.5 ml reaction tubes. Forty-five μl of dinitrophenylhydrazine (DNP) solution [35] was added to a 15 μl sample. A final protein concentration was 1 mg/ml. Absorbances were read with a 490 nm filter using a micro plate reader. The absorbance of the blank sample in PBS without protein was subtracted from all other absorbances. A seven-point standard curve of oxidized BSA (Sigma, Germany) was included with each plate [34].

### Blood chemistry

Plasma glucose was analyzed enzymatically with hexokinase (Olympus Diagnostica, Hamburg, Germany) and serum insulin by radioimmunoassay (Pharmacia, Uppsala, Sweden). Hemoglobin A1c (HbA_1c_) concentration was assayed by using the latex immunoagglutination inhibition method (Bayer Corporation, Elkhart, IN, USA: DCA, 2000 Reagent Kit). Insulin resistance was determined by the homeostasis model assessment for insulin resistance (HOMA-IR) as described by Matthews et al. [36]. Serum uric acid was determined photometrically by the hydroxylamine method [37]. Oxygen radical absorbing capacity (ORAC) was assayed by using a multi-well plate reader according to the methods described elsewhere [38,39]. Briefly, the antioxidant capacity of the samples was measured by the inhibition of the decrease of the fluorescence. For this purpose, fluorescein was used as a target of free radical attack, with 2,2'-azobis (2-amidinopropane) dihydrochloride as a peroxyl radical generator.

### Statistical analysis

Data are reported as means ± standard error (SE). Student's paired t test was used to assess differences within groups (baseline and 2-year follow-up) and Kruskall-Wallis test for difference between the groups. Pearson's correlation coefficients were used to express the associations between the variables.

## Results

### Glucose homeostasis, aerobic capacity, oxidative stress and its defence mechanisms in subjects with IGT

Baseline and 2-year follow-up values of the IGT subjects (n = 22) are shown in Table 2. Body weight decreased by -4.4 kg (4.9%) (*P *< 0.001). In addition, the fasting glucose, 2-h glucose, HbA_1c_, serum protein carbonyl and uric acid concentrations decreased significantly in the IGT subjects (Table 2). Maximal oxygen uptake variables (VO_2max _and VO_2max_/weight) and the protein content of GRP75 and HSP60 increased significantly, compared with the pre-intervention values (Table 2).

**Table 2:** Indices of glucose m etabolism, weight, m axim al oxygen uptake and oxidative stress in the subjects with im paired glucose tolerance (IGT), n = 22.

	Baseline	2-year follow-up	Change	*P *value
Fp-glucose, m m ol/l	6.0 ± 0.1	5.8 ± 0.1	-0.3 ± 0.1	0.013
2h-glucose, m m ol/l	7.7 ± 0.3	6.4 ± 0.4	-1.2 ± 0.5	0.013
HbA_1c_, %	5.7 ± 0.1	5.4 ± 0.1	-0.4 ± 0.1	<0.001^a^
W eight, kg	89.7 ± 1.8	85.3 ± 2.0	-4.4 ± 1.1	<0.001
BM I (kg/m^2^)	29.9 ± 0.5	28.4 ± 0.5	-1.5 ± 0.4	<0.001
VO_2max_, l/min	2.28 ± 0.14	2.43 ± 0.13	0.2 ± 0.0	<0.001
VO_2max_/weight, ml/kg/min	26.1 ± 1.6	28.5 ± 1.5	2.4 ± 0.6	<0.001
GRP75 (muscle), arb. u.	0.95 ± 0.11	1.28 ± 0.16	0.33 ± 0.12	0.014
HSP60 (muscle), arb. u.	0.35 ± 0.05	0.56 ± 0.11	0.21 ± 0.08	0.022
P. carbonyls (serum), arb. u.	0.12 ± 0.00	0.11 ± 0.00	-0.00 ± 0.00	0.010^b^
Uric acid, μmol/l	375 ± 12	337 ± 14	-37 ± 9	0.001

Data are given as m eans ± SE; arb. u. = arbitrary units, ^a ^n = 21, ^b ^n = 20

### Comparison of IGT sub-groups

At baseline, HbA_1c _was similar in the IGTslow and IGTfast group, while HOMA-IR was 49% (1.7 units) higher (*P *= 0.087) in the IGTfast group (Table 3). Most of the measured markers of oxidative stress and antioxidative capacity of serum and skeletal muscle were similar between the groups. The serum protein carbonyl concentration was, however 17% higher in the IGTslow than in the IGTfast group (*P *= 0.002, Table 3). The total energy intake and vitamin C were similar at baseline, but the vitamin E intake was 34% (4.2 mg/day) lower in the IGTslow than in the IGTfast group (*P *= 0.003).

**Table 3:** Indices of glucose m etabolism and oxidative stress from serum and skeletal m uscle sam ples, as well as m axim al oxygen uptake in the subjects with im paired glucose tolerance (IGT) divided into two muscle phenotype groups.

	IGTslow subjects Baseline	2-year follow-up	IGTfast subjects Baseline	2-year follow-up
*n *= (Female/Male)	10 (4/6)		12 (2/10)	
*Serum*				
HbA_1c_, %	5.74 ± 0.13	5.38 ± 0.08*	5.67 ± 0.11	5.33 ± 0.14**
HOM A-IR	3.52 ± 0.30	3.22 ± 0.51	5.23 ± 0.75	3.77 ± 0.71*
ORAC, μmol	56.0 ± 4.5	58.2 ± 3.1	54.0 ± 3.4	55.4 ± 3.2
P. carbonyl, arb.u.	0.129 ± 0.005	0.126 ± 0.00	0.110 ± 0.003^##^	0.106 ± 0.003* ^##^
Uric acid, μmol/l	370 ± 17	331 ± 18**	379 ± 17	342 ± 21*
				
*Skeletal muscle*				
P. carbonyl, arb.u.	0.074 ± 0.011	0.071 ± 0.008	0.072 ± 0.007	0.070 ± 0.005
4-HNE, arb.u.	0.29 ± 0.07	0.24 ± 0.04	0.25 ± 0.04	0.26 ± 0.04
				
*Maximal oxygen uptake*				
VO_2max_, l/min	1.96 ± 0.22^a^	2.10 ± 0.20* ^a^	2.52 ± 0.15	2.69± 0.15**
VO_2max_, /weight ml/kg/min	23.3 ± 2.7^a^	25.3 ± 2.4* ^a^	28.2 ± 1.7	30.9 ± 1.6**

Data are given as means ± SE; *P < 0.05, ** *P *< 0.01 within group; ^#^*P *< 0.05, ^##^*P *< 0.01 between groups;^a ^*n *= 9.

**Figure 1:**
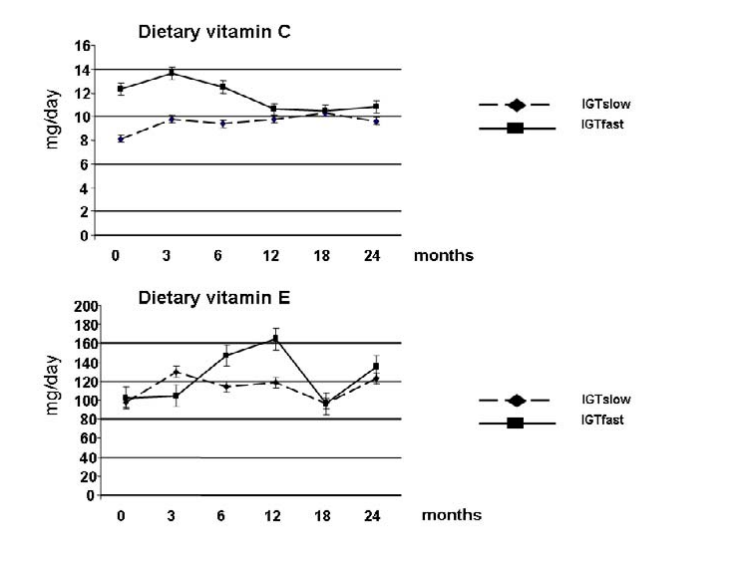
Dietary vitamin C and E intakes during a 2-year intervention in subjects with impaired glucose tolerance (IGT), divided into slow (n = 10) and fast (n = 12) fibre type sub-groups.

During the 2-year intervention, there were no significant changes in the energy intake or vitamin C in the IGTslow and IGTfast groups. Vitamin E intake was lower in the IGTslow than in the IGTfast group during the whole study period (Figure 1). During the follow-up period, body weight reduced by 5.1 (SD 6.2; range: 0.3 to -9.9) kg in the IGTfast group and by 3.6 (SD 3.2; range: 3.0 to -14.5) kg in the IGTslow group [24]; but the difference between the groups was not statistically significant.

Maximal oxygen uptake variables increased and HbA_1c _concentrations decreased in both groups but HOMA-IR values decreased only in the IGTfast group (Table 3). Serum concentrations of uric acid decreased significantly in both groups (Table 3), but serum protein carbonyl concentrations decreased only in the IGTfast group (*P *= 0.0213). After 2 years, the serum protein carbonyl concentrations were still higher in the IGTslow than in the IGTfast group (*P *= 0.015) (Table 3). The intervention period did not significantly affect the ORAC values in either group (Table 3). There were no changes in protein oxidation or lipid peroxidation in the skeletal muscle in either group (Table 3).

### Chaperone responses

**Figure 2:**
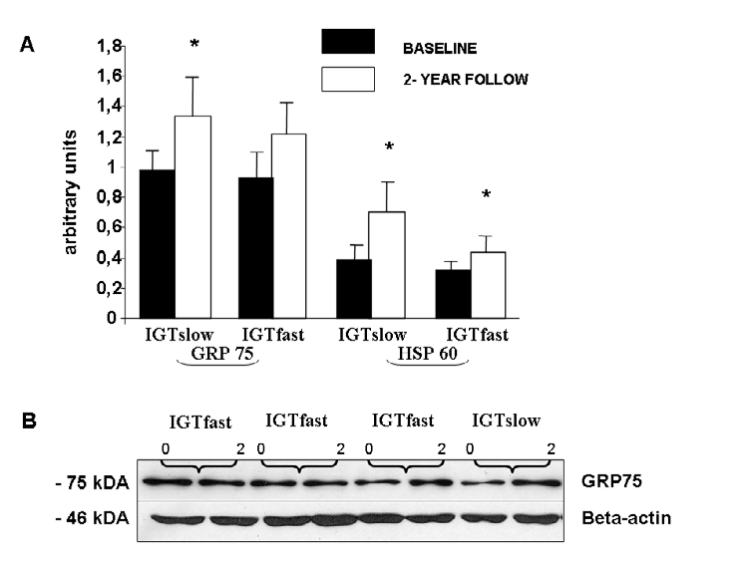
**(A) Effects of the 2-year intervention on the protein expression of GRP75 and HSP60 in the *vastus lateralis *muscle of subjects with impaired glucose tolerance (IGT), divided into slow (n = 10) and fast (n = 12) fibre type sub-groups.** Data are given as means ± SE. *P < 0.05 within groups. (B) Representative Western blots, using anti-GRP75 and beta-actin antibodies, of whole tissue homogenates from skeletal muscle biopsies of four IGT subjects at baseline (0) and after 2-year follow-up (2).

**Figure 3:**
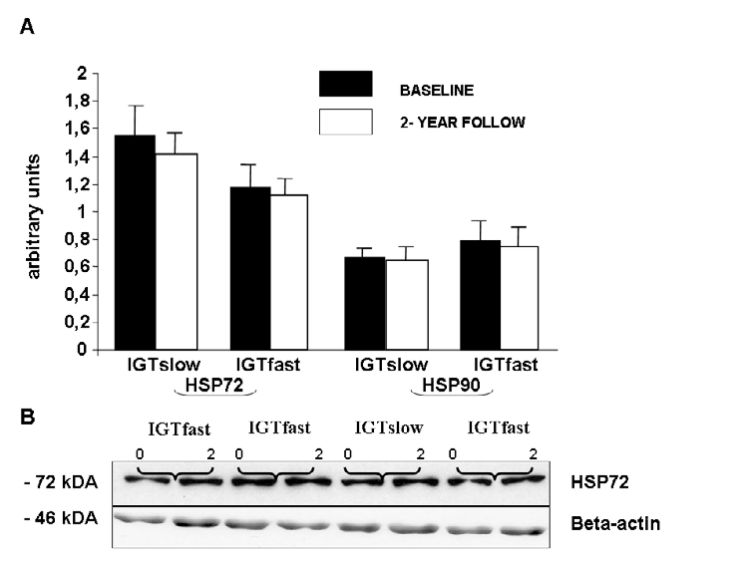
**(A) Effects of the 2-year intervention on the protein expression of HSP72 and HSP90 in the *vastus lateralis *muscle of subjects with impaired glucose tolerance (IGT), divided into slow (n = 10) and fast (n = 12) fibre type sub-groups.** Data are given as means ± SE. *P < 0.05 within groups. (B) Representative Western blots, using anti-HSP72 and beta-actin antibodies, of whole tissue homogenates from skeletal muscle biopsies of four IGT subjects at baseline (0) and after 2-year follow-up (2).

During the intervention, the expression of mitochondrial HSP60 increased (79% in the IGTslow and 38% IGTfast group) significantly in both groups (IGTslow: *P *= 0.034 and IGTfast: *P *= 0.029) and GRP75 increased (38%) significantly in the IGTslow group (*P *= 0.022), while only a tendency was recorded in the IGTfast group (*P *= 0.072; Figure 2). On the other hand, no changes were observed in the cytoplasmic chaperones HSP72 or HSP 90 in either of the groups (Figure 3).

**Figure 4:**
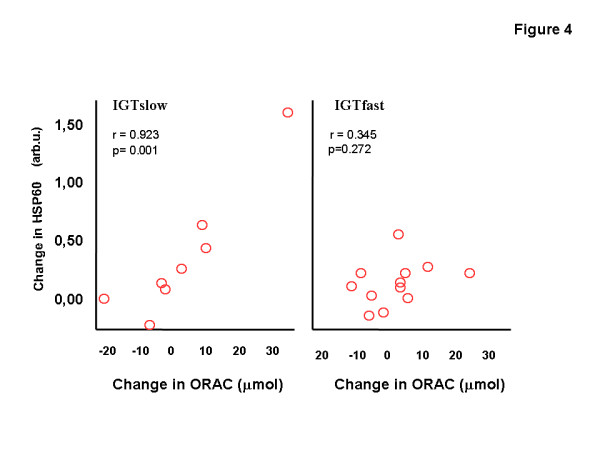
Relationship between the changes of the HSP60 and ORAC values during a 2-year intervention in subjects with impaired glucose tolerance (IGT), divided into slow (n = 8) and fast (n = 12) fibre type sub-groups.

**Figure 5:**
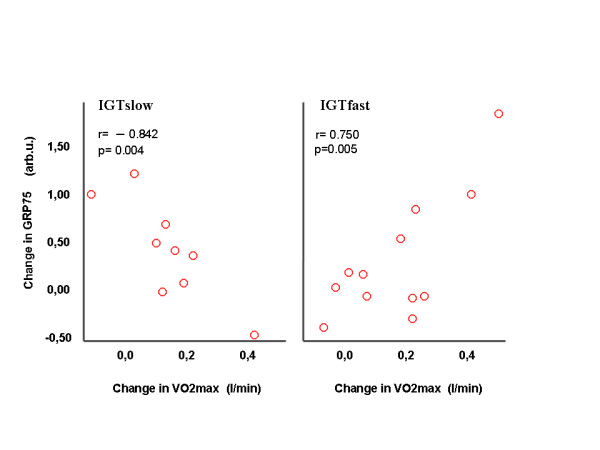
Relationship between the changes of the GRP75 and VO_2max _values during a 2-year intervention in subjects with impaired glucose tolerance (IGT), divided into slow (n = 9) and fast (n = 12) fibre type sub-groups.

There were positive correlations between the increase of muscle HSP60 and the increase of the serum ORAC values (*r *= 0.92; *P *= 0.001) in the IGTslow group (Figure 4). The improvement of maximal oxygen uptake (VO_2max_/weight) correlated negatively with decreased weight (*r *= -0.617; *P *= 0.033) and 2-h glucose (*r *= -0.613; *P *= 0.034) and the improved VO_2max _value correlated positively with the increase of protein expression of GRP75 (*r *= 0.750; *P *= 0.005) in the IGTfast group and negatively in the IGTslow group (r = -0.842; P = 0.004) (Figure 5).

## Discussion

This is the first study to investigate the HSP responses in human skeletal muscle tissue during a long-term exercise-diet intervention. After the intervention period, oxidative stress was reduced as shown by decreased serum levels of uric acid and protein carbonyls, and cytoprotection was improved in the skeletal muscle tissue, observed as the increased expression of mitochondrial HSP60 and GRP75 in the IGT subjects while no response was found in cytoplasmic chaperones HSP72 and HSP90.

In this study, we aimed to investigate if the muscle fibre composition is associated with the above improvements, and hence the IGT subjects were into two sub-groups (IGTfast and IGTslow). In general, men and women have, on an average, 45% and 55% of MHC I fibers (slow oxidative fibers) [40,41]. In our study, the muscle fibre composition of the two sub-groups differed significantly [24], The fibre type composition remained unchanged in the two subject groups, although the exercise training slightly increased (n.s) the proportion of MHC I and decreased (n.s) the proportion of MHC IIx isoforms in the *vastus lateralis *muscle in IGTslow group [24]. Otherwise, there were no differences between the sub-groups in physical activity or in dietary intake before or during the intervention, except for vitamin E intake at baseline.

### Chaperones and exercise

Physical inactivity is one of the major non-genetic determinants of type 2 diabetes. In the present study, the improvement of maximal oxygen uptake in both sub-groups shows that the exercise training protocol was effective. Presumably, the increased expression of mitochondrial HSP60 and GRP75 in *vastus lateralis *muscle in the IGTslow group and that of HSP60 in the IGTfast group were responses to our intervention program. Induced HSP60 and HSP72 expressions in skeletal muscle have also been reported in a short-term exercise intervention in healthy humans [42,43]. Consistently, 8 weeks of endurance training increased GRP75 expression in red *gastrocnemius *muscle of streptozotocin-induced diabetic (SID) rats as well as non-diabetic control rats [2]. In addition, we have recently shown that after four weeks of immobilization, acute intensive non-damaging exercise up-regulated especially HSP60 expression in the *plantaris *and *gastrocnemius *muscles in rats [32]. Furthermore, Mattson et al. [44] have reported that both HSP60 and GRP75 are up-regulated in rat skeletal muscle after 8 weeks of endurance training. In those studies, up-regulation of HSP60 and GRP75 was associated with increased skeletal muscle citrate synthase activity (CS) [2,44]. Menshikova et al. [45] have recently shown that a 4 month walking combined with weight-loss increased CS activity 29 ± 9% in previously sedentary obese men and women and induced mitochondrial biogenesis in skeletal muscle. Our exercise program also increased CS activity in *vastus lateralis *muscle in both groups (34% in the IGTslow and 20% IGTfast group) [24]. However, we did not find any correlation between increased CS activity and HSP60 or GRP75 expression. On the other hand, the improvement in VO_2max _correlated positively with increased protein expression of GRP75 in the IGTfast group but not with the weight loss.

Diabetes has been reported to cause mitochondrial dysfunction and attenuated synthesis of a variety of proteins, including HSP60 in mitochondria [46,47]. The skeletal muscle insulin resistance and mitochondrial dysfunction are associated with ageing, type 2 diabetes and in offspring of type 2 diabetes [48]. The reduced oxidative activity could also be a result of a sedentary lifestyle, and no data on correlations between changes in oxidative capacity and insulin sensitivity following exercise in type 2 diabetes have been published [49]. The capacity of HSP60 to stabilize mitochondrial proteins, promote mitochondrial protein biosynthesis and to prevent mitochondrial apoptosis seems to be crucial for its protective function [50]. In the present study, the adaptive changes in the expression of GRP75 and HSP60 could support mitochondrial protein import, protein folding and enhance tissue protection in insulin-resistant muscle tissue. These adaptive changes of mitochondrial HSPs and increased oxidative capacity are mainly due to increased contractile activity of skeletal muscle. However, Civitarese et al. [51] have recently shown in young obese subjects that six months caloric restriction alone, or with exercise reduces DNA damage and increases muscle mitochondrial biogenesis (up regulation of genes involving mitochondrial function) without any increase of oxidative enzymes including CS. Skeletal muscle from obese subjects contains a smaller number of mitochondria because of the lower ratio of type 1 to type 2 muscle fibers and have reduced oxidative capacity [45,52]. Several genes acting in antioxidative processes against oxidative stress are defective in diabetic subjects and correlate with the oxidative capacity [19,52]. Exercise activates PGC-1α, which mainly mediates transcriptional regulation of nuclear genes encoding mitochondrial proteins (NGEMP). These nuclear-derived gene products have to import into mitochondria via the protein-import machinery. Imported NGEMPs interact with GRP75, and GRP75 drives translocation of these precursors across the mitochondrial matrix. In an ATP-dependent process precursors are released and bound to the HSP60. Therefore, both GRP75 and HSP60 are essential for mitochondrial function and biogenesis [17]. Also, it has recently been shown that the basal expression of HSP72 mRNA in the skeletal muscle is lower in type 2 diabetics, compared with healthy control subjects, which may lead to an imbalance of antioxidant defence mechanism [19].

In the present study, interestingly the expression of the cytoplasmic HSP72 and HSP90 in skeletal muscle was not affected by the exercise-diet intervention in either of the study groups. Most studies showing that exercise induced HSP72 [19], have used low force exercises with continuous activity. However, Gjovaag et al. [53] have shown that high force exercise with intermittent activity like weight training decrease HSP72 expression in very well trained males. In this study, we used similar type of supervised strength and power-type exercise than Gjovaag et al., and it may be one reason why our exercise program did not increase the expression of the HSP72. On the other hand since HSP induction is also dependent on the training levels of the subjects prior to the exercise intervention [54,55], we may not directly compare the responses of the sedentary subjects with very well trained ones.

Furthermore, we aimed to investigate the association of tissue defences with improved glucose metabolism. We found a decrease of the 2-h glucose and HbA_1c _concentrations in the IGTslow and IGTfast groups and a decrease of the fasting glucose and HOMA-IR values in the IGTfast group [24]. These results also agree with those presented in a meta-analysis by Snowling and Hopkins [56] showing that aerobic, resistance, and combined exercise have small to moderate beneficial effects on glucose metabolism in type 2 diabetic subjects. However, our findings did not correlate with the markers of improved cytoprotection of the skeletal muscle in either group. Fasting values of glucose and insulin (HOMA-IR) are depending on the hepatic glucose control, but not peripheral glucose metabolism. Petersen et al. [57] have show that improvements in basal and insulin-stimulated hepatic glucose metabolism were associated reduction in intrahepatic lipid (IHL) (P = 0.0009) without significant changes in insulin-stimulated peripheral glucose uptake after a moderate weight loss (8% of their body weight). The improvement of the VO_2max_/weight correlated negatively with the 2-h glucose values at the 2-year follow-up in the IGTfast group.

### Antioxidant capacity

In addition to the HSP responses, we studied the antioxidant capacity as measured by ORAC in the serum. ORAC concentrations were slightly increased in both study groups during the intervention but the increase was not statistically significant. In agreement with our results, a combination of antioxidant mixture supplementation and 24 days of cold-weather field training did not significantly affect ORAC or other oxidative stress markers [4]. On the other hand, changes in the serum levels may not always reflect the trainings adaptations gained in skeletal muscle, especially if the samples were taken at rest. We, however, found a significant positive correlation between the increased content of HSP60 and the ORAC values in the IGTslow group. No study has so far been published investigating the association between the ORAC and HSP levels. In studies applying other methods for antioxidant capacity have been used, endurance training contributed to the improved cardiac protection by enhancing the antioxidant capacity and HSP induction [54,55].

No association was found between the improvements in the glucose metabolism and the antioxidant capacity or HSP defence during the intervention, although it has been recently shown that, in type 2 diabetic subjects, insulin resistance correlates with decreased expression of HSP72 in skeletal muscle at mRNA level [6].

### Oxidative stress

Increased production of ROS is typical in type 2 diabetic subjects [18,58]. Hyperglycemia decreases glutathione synthesis and impairs the antioxidant defence [17,59]. One of the most toxic aldehydes formed during lipid peroxidation is 4-hydroxynonenal (HNE), which can denature proteins and form protein adducts. It has been demonstrated that the induction of HSP72 is closely associated with and regulated by 4-HNE protein adducts [60]. In the present study, however, we neither observed any differences in the HSP72 expression nor 4-HNE protein adduct levels in the skeletal muscles of the subjects with impaired glucose tolerance. The only previously published study has shown that both long-term exercise training and one week of intensive resistance training resulted in increased 4-HNE, malondialdehyde (MDA) and thiobarbituric acid-reactive substance (TBARS) levels in female weightlifters [61]. The intensity of exercise in the present study was not as high as in the Liu's study [61]. Protein carbonyls have been widely used marker of oxidative modification of proteins. Elevated protein carbonyl levels have been detected both in type 1 and type 2 and also in experimental diabetes [2,15,62]. We observed a small, but statistically significant decrease of plasma protein carbonyls after the intervention in the IGTfast group. In agreement with our results, a recent study showed that 14 weeks of training decreased protein carbonyl content and induced HSP60 expression without affecting HSP72 expression in the heart of doxorubicin treated mice [63]. Furthermore, moderate training decreased the protein carbonyl content in rat liver without affecting lipid peroxidation [64].

## Conclusion

To conclude, exercise training combined with dietary counselling improved glucose metabolism and maximal oxygen uptake in subjects with impaired glucose tolerance, regardless of the muscle fibre phenotype. The intervention increased the mitochondrial chaperones in the skeletal muscle, while no changes took place in the cytoplasmic chaperones. The mitochondrial responses in the IGTslow sub-group were slightly greater than in the IGTfast subgroup. The enhancement of mitochondrial HSP defences may reduce glucose toxicity, since muscle mitochondria are important sources of oxygen derived radicals and since insulin resistance and diabetes are associated with increased oxidative stress.

## Abbreviations

DPS: the Finnish Diabetes Prevention Study; GRP75: glucose-regulated protein 75; HbA_1c_: Hemoglobin A1c; HNE: 4-hydroxynonenal; HOMA-IR: homeostasis model assessment for insulin resistance; HSP: heat shock protein; IGT: impaired glucose tolerance; IHL: intrahepatic lipid; MAPK: mitogen-activated protein kinase; MHC: myosin heavy chain; NGEMP: nuclear genes encoding mitochondrial proteins; ORAC: oxygen radical absorbing capacity; PKB/AKT: protein kinase B; ROS: reactive oxygen species; SID: streptozotocin-induced diabetes TBARS: thiobarbituric acid-reactive substance; VO_2max_: maximal oxygen uptake.

## Competing interests

The author(s) declare that they have no competing interests.

## Authors' contributions

HH, JM and SA were responsible overall design of study. MV, SA, MA and MG were responsible of the analysis of the results and MV and SA for statistical analysis. MG performed most of the biochemical analyses and MA and MV provided technical guidance and advice for biochemical methods. RP was performed MHC analysis. JPH was responsible of taking off muscle biopsies. JL and MR were responsible of dietary intervention and analysis. KH, OH, and PN critically reviewed the manuscript, with important impact of intellectual content. MV wrote the draft of the manuscript. MA and SA provided the critical revision of the manuscript. All the authors have read and approved the final manuscript.

## Pre-publication history

The pre-publication history for this paper can be accessed here:


